# Applications of yoga in oral oncology: A systematic scoping review

**DOI:** 10.1002/hsr2.1208

**Published:** 2023-04-11

**Authors:** Kehinde K. Kanmodi, Ramat O. Braimah, Jimoh Amzat, Afeez A. Salami, Lawrence A. Nnyanzi

**Affiliations:** ^1^ School of Health and Life Sciences Teesside University Middlesbrough UK; ^2^ Faculty of Dentistry University of Puthisastra Phnom Penh Cambodia; ^3^ Campaign for Head and Neck Cancer Education (CHANCE) Programme Ibadan Nigeria; ^4^ Department of Oral and Maxillofacial Surgery, Faculty of Dental Sciences Usmanu Danfodiyo University Sokoto Nigeria; ^5^ Department of Sociology Usmanu Danfodiyo University Sokoto Nigeria; ^6^ Department of Sociology University of Johannesburg Johannesburg South Africa; ^7^ Department of Oral and Maxillofacial Surgery University College Hospital Ibadan Nigeria

**Keywords:** meditation, oral cancer, scoping review, Yoga therapy

## Abstract

**Background and Aims:**

Yoga is well‐thought‐out as an all‐inclusive approach globally and can be administered in clinical care as an integrative or alternate approach to regular treatment. Yoga exercise has been disclosed to influence remission from cancer cells over a long period of time and also reverses epigenetic alterations. Applications of Yoga in the management of oral oncology patients are scarce, hence the need for a scoping review of the literature. Hence, this study aimed to conduct a scoping review of the existing empirical evidence on the applications of yoga in oral oncology.

**Methods:**

The review methodology was informed by Joanna Brigg's Institute guidelines for systematic scoping reviews, and the review was reported in accordance with the Preferred Reporting Items for Systematic Reviews and Meta‐Analyses extension for Scoping Reviews. Ten databases were searched. The records of all the literature retrieved from the search were imported into the Rayyan software for deduplication. After the full‐text screening, only two were found eligible for inclusion in the scoping review. Data obtained in the included literature were extracted and synthesized.

**Results:**

This review found that Yoga was not significantly effective in the management of stress among oral cancer patients (*p*‐values > 0.04). However, it was found that Yoga significantly reduced anxiety, saliva stickiness, and episodes of falling ill (*p*‐values < 0.05) while it improved mental well‐being, cognitive functioning, emotional functioning, and head and neck pain of those oral cancer patients that received it (*p*‐values < 0.05).

**Conclusion:**

An integrative care approach that considers nonpharmaceutical techniques such as yoga could help to reduce care cost while improving care outcomes and quality of life of oral cancer patients. Hence, it is imperative to consider yoga along with its potential benefits, and we recommend gradual incorporation of yoga into oral cancer care.

## 1 INTRODUCTION

Oncology is the discipline of medicine that specializes in the diagnosis and management of cancers in the body, according to the US National Cancer Institute.^1^ Cancer is a disorder whereby specific body's cells propagate hysterically and disseminate to other body parts. Oral cancer has been placed third globally after breast and lung cancers and within the head and neck region as the most frequent type of cancer.^2^, ^3^, ^4^

Head and neck cancer, which includes the oral cancer, is the seventh commonest cancer worldwide, estimating for more than 660,000 fresh cases and 325,000 mortalities every year.^5^ In the Asian continent, oral cancer is among the commonest types of cancers because of the high tobacco usage, betel quid chewing and excessive alcohol intake. Within Asia, prevalence likewise differs whereby the highest frequency was observed in Southern Asia exclusively in countries such as Bangladesh, Afghanistan, Sri Lanka, India, and Pakistan.^6^ Europe is placed second after Asia in terms of incidence of oral cancer and rates also vary within the region.^7^ In North America (Canada) and South America (Mexico), oral cancer is ranked as the twelfth and thirteenth most common cancer respectively, while in the United States, it is ranked the eleventh.^8^ These global reports have shown consistently increasing trend in the frequency of oral cancer despite various interventions at the communal and hospital care levels by the authorities and nongovernmental agencies.^9^

Regardless of evasion of disease‐specific chance and dynamics as preventive measures, management strategies are also developing innovative techniques of improving quality of life of oral cancer patients.^10^ Currently, surgery is regarded as the best approach for the management of oral cancer,^4^, ^11^ with adjuvant or neo‐adjuvant chemo‐radiation to eliminate residual tumor or down‐stage tumor before surgery.^12^, ^13^ Despite the combination of all preventive and treatment strategies, the quality of life of oral cancer patients still remains deprived.^14^ This is because various factors affecting quality of life in oral cancer patients has not been adequately addressed by conventional preventive and management modalities. These factors include physical factors such as over‐all well‐being, discomfort, sleep quality and quantity, anxiety, exhaustion, and speech quality.^15^, ^16^ Other ancillary unaddressed factors may involve economic strains, issues with family support, protracted treatment phase, numerous clinic appointments, and illness relapse.^17^

Despite all the powerful technologies and strong targeted medications for cancer patients which are very costly and associated with lethal side effects, the desired success still remains elusive for modern medicine.^14^ Patients consequently may turn to nonconventional treatments such as complementary and alternative medicine (CAM).^18^

Yoga, which comprises of a wide range of exercises is an element of CAM which steadily harmonizes the body and mind.^19^ Yoga is well‐thought‐out as an all‐inclusive approach globally which can be administered in medical care as a complementary or alternative approach to routine care.^20^ Studies has shown that the practice of Yoga asanas, meditation and pranayamas can cutback carcinogenesis.^21^ Additionally, Yoga exercise has been disclosed to positively impact on the long‐term remission from cancer cells and it also helps reverses epigenetic alterations by DNA methylation, histone modifications, miRNAs and epi‐transcriptomics.^22^, ^23^ Epigenetics which was first coined by Conrad Waddington in 1940 has undergone extensive study and mainly involves alterations in transcriptional expression and/or activity without variation in DNA sequence.^23^

Applications of Yoga in the management of oral oncologic patients is scarce, hence the need for a scoping review of the literature. Consequently, the key aim of this scoping review is to find out about existing literature on applications of Yoga in oral oncology management.

## 2 METHODS

The Joanna Briggs Institute's guidelines for systematic scoping reviews (ScRs) informed the design of this ScR.^24^ Also, the reportage of this ScR was informed by the Preferred Reporting Items for Systematic Reviews and Meta‐Analyses extension for Scoping Reviews (PRISMA‐ScR) checklist.^25^ This ScR seeks to provide answers to this principal question: what are the existing empirical evidence on the applications of Yoga in oral oncology?

To address this question, we conducted a systematic search of 10 electronic databases (PubMed, SCOPUS, AMED, CINAHL Complete, CINAHL Ultimate, Dentistry and Oral Sciences Source, SPORTDiscus with Full Text, APA PsycInfo, APA PsycArticles, and Psychology and Behavioral Sciences Collection), on the January 27, 2023, to identify literatures relevant to the question using a combination of the terms “yoga,” “oral cancer” and their synonyms, and with the aid of Boolean operators (“OR” and “AND”) and truncation (“*”). The search strings generated from these search combinations are depicted in Tables A1, A2, A3 (Appendix).

The records of all the literature retrieved from the scoping search were imported into the Rayyan software for deduplication.^26^ All duplicate records were removed. Thereafter, the deduplicated literatures were screened to exclude those literatures which are not relevant to the ScR question. The screening was done by two independent researchers, two‐staged, and it was guided by a set of selection criteria. The first stage of the screening involved title and abstract screening while the second stage involved full text screening.

Only those literatures that met all of the following criteria were included in the ScR: peer‐reviewed journal papers; papers published in English; papers reporting empirical findings on the application of yoga in the field of oral oncology; and papers with accessible full texts. However, all those literatures that do not meet all the above criteria were excluded from the ScR.

From the included literature, data were extracted using a customized data extraction sheet (Table 1). The extraction sheet obtained information on the author names, year of publication, journal of publication, country where the study was conducted, sample characteristics, study design, study instrument, study outcomes, and conclusions. Only qualitative data synthesis was done on the extracted data—this involved the collation and summarization of the extracted data in a prose format. In this synthesis, relevant statistics were reported using mean (M), standard deviation (SD), and *p*‐values, with a *p*‐value < 0.05 considered to be of statistical significance. Quantitative data analysis (i.e., meta‐analysis) was intended to be done; however, this could not be done due to the multiple heterogeneities in the methodology (research design, population characteristics, data analysis approach) of the included articles.^28^

**Table 1:** Data extraction sheet.

S/N	Author	Year of publication	Journal	Objectives	Study design	Country	Sample characteristics	Study Instrument	Conclusion
1	Bakshi & Goyal^20^	2021	Sport Sciences for Health	To investigate the role of yoga in improving the quality of life of advanced‐stage oral cancer patients.	Randomized blinded trial	India	Twenty‐six advanced‐stage oral cancer patients (12 were lost to follow up); 23 (88.46%) were males. No information was provided on participants' age, culture, and religion.	Questionnaires (EORTIC‐H&N‐35; Voice Handicap Index [VHI]; General Health Questionnaire [GHQ‐12]; Perceived Stress Scale [PSS]; Warwick‐Edinburgh Mental Well‐being Scale [WEMWBS]); digital semi‐automated blood pressure measurement device.	The integrative administration of yoga with conventional treatment can help to improve the quality of life of oral cancer patients.
2	Pattnaik et al.^27^	2020	Journal of Family Medicine and Primary Care	To evaluate the impact of yoga on oral cancer and stress management.	Prospective study	India	Two hundred oral cancer patients. No information was provided on participants' age, culture, and religion.	Customized questionnaire.	Yoga is effective in the management of stress among oral cancer patients.

## 3 RESULTS

**Figure 1:**
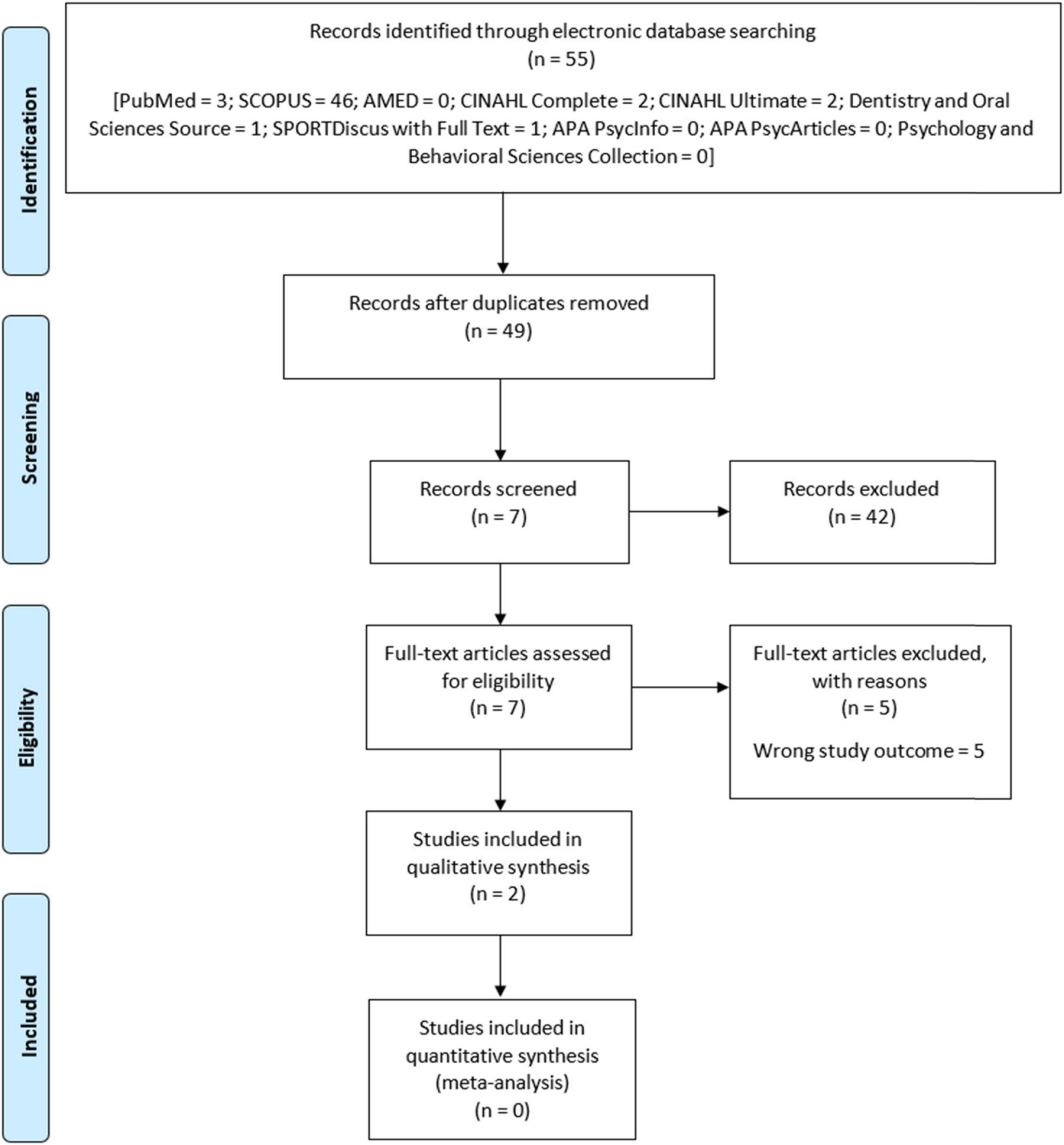
Flow chart diagram.

From the 10 electronic databases searched, a total of 55 publications were retrieved. From these publications, six were duplicated copies and were all removed. After the screening of the titles and abstracts of the remaining 49 publications, 42 publications were excluded. After the full text screening of the remaining seven publications, only two publications were finally included into the scoping review (Figure 1; Table A4 [Appendix]).

### 3.1 Country location, and authors, journals and years of publication of studies

The two studies were conducted in India, and had at least two authors, all of which were affiliated to institutions in India.^20^, ^27^ One was published in 2020 while the other was published in 2021. One was published in Sport Sciences for Health while the other was published in Journal of Family Medicine and Primary Care.^20^, ^27^

### 3.2 Study design

Both studies were intervention‐based.^20^, ^27^ However, the study by Bakshi and Goyal^20^ adopted a randomized blinded trial design and with two compared groups while the other study, by Pattnaik et al.,^27^ was branded a prospective study with no compared group, that is, all the participants in the study were exposed to same intervention (yoga therapy).

### 3.3 Sample characteristics

All the participants recruited into both studies were oral cancer patients.^20^, ^27^ None of the two studies reported data on the age distribution, culture, and religion of the participants. Also, only one study (by Bakshi and Goyal^20^) reported data on the sociodemographic characteristics of their participants; the information included sex, locality, educational status, employment status, monthly family income, and addiction history.

### 3.4 Duration of yoga intervention

The intervention in Bakshi & Goyal's study was a yoga therapy delivered in two phases: Phase 1 (before surgery) for a duration of 2 weeks; and Phase 2 (after wound healing has been achieved post‐surgery) for a duration of 2 months.^20^ However, in the study by Pattnaik et al.,^28^ the intervention was a 1‐month yoga training.

### 3.5 Themes investigated and study instruments

The study by Bakshi and Goyal^20^ investigated the role of yoga in improving the quality of life of advanced‐stage oral cancer patients while the study by Pattnaik et al.^27^ evaluated the impact of yoga on oral cancer and stress management. Bakshi and Goyal's study used multiple standardized questionnaires and digital semi‐automated blood pressure measurement device (DSBPMD) for data collection. The questionnaires used were EORTIC‐H&N‐35, Voice Handicap Index [VHI], General Health Questionnaire [GHQ‐12], Perceived Stress Scale [PSS], and Warwick–Edinburgh Mental Well‐being Scale [WEMWBS], and they were used to assess their participants' overall quality of life, voice quality, general health, stress and anxiety level and mental well‐being, respectively. The DSBPMD was used to measure the participants' blood pressure.

The study by Pattnaik et al.^27^ used a customized questionnaire, of which its validity and reliability status were not mentioned. The questionnaire obtained information about the experiences and feelings of the participants concerning anxiety, apprehension, nightmares, sleep interruption, fear levels, social stigma, and yoga therapy.

### 3.6 Impact of yoga on the participants

Both studies reported that yoga interventions had some impacts on some domains of health of oral cancer patients.^20^, ^27^ The domains were indicated below.

#### 3.6.1 Quality of life

Only one study, by Bakshi and Goyal,^20^ explored the role of yoga therapy on the quality of life of oral cancer patients, and this was done using the EORTIC‐H&N‐35 questionnaire. In the study, an insignificant increase in the overall quality of life of the control group (baseline data ‐ 89.39 (M) ± 29.13 (SD); endline data ‐ 138.89 (M) ± 50.92 (SD); *p*‐value = 0.074) and experimental group (baseline data ‐ 89.39 (M) ± 29.13 (SD); endline data ‐ 125 (M) ± 36.13 (SD); *p*‐value = 0.107); however, the level of increase was reportedly lower among those that received yoga compared with those patients who received it not. Furthermore, from the exploration of the various subdomains of quality of life explored, only the subdomains of cognitive functioning (baseline data [all participants] ‐ 93.94 [M] ± 32.72 [SD]; endline data [control group] ‐ 77.78 [M] ± 19.24 [SD], *p*‐value = 0.601; endline data [experimental group] ‐ 55.56 [M] ± 17.22 [SD], *p*‐value = 0.026), emotional functioning (baseline data [all participants] ‐ 342.4 [M] ± 117.5 [SD]; endline data [control group] ‐ 166.7 [M] ± 66.67 [SD], *p*‐value = 0.043; endline data [experimental group] ‐ 200 [M] ± 101.1 [SD], *p*‐value = 0.035), head and neck pain (baseline data [all participants] ‐ 333.3 [M] ± 89.44 [SD]; endline data [control group] ‐ 244.4 [M] ± 69.39 [SD], *p*‐value = 0.232; endline data [experimental group] ‐ 222.2 [M] ± 83.44 [SD], *p*‐value = 0.039), saliva stickiness (baseline data [all participants] ‐ 123.5 [M] ± 91.87 [SD]; endline data [control group] ‐ 66.67 [M] ± 51.64 [SD], *p*‐value = 0.175; endline data [experimental group] ‐ 16.67 [M] ± 26.59 [SD], *p*‐value < 0.001), and falling ill (baseline data [all participants] ‐ 121.6 (M) ± 92.75 (SD); endline data [control group] – 0 [M] ± 0 [SD], *p*‐value = 0.001; endline data ‐ 8.33 (M) ± 15.07 (SD), *p*‐value < 0.001]) were significantly improved through yoga therapy, and the degree of improvements observed in these domain were higher than those observed among those who did not receive yoga therapy (i.e., the control group). However, no significant improvement or worsening of other sub‐domains in the EORTIC‐H&N‐35 questionnaire were recorded in the study.^20^

#### 3.6.2 Voice quality

Only one study, by Bakshi and Goyal,^20^ explored the impact of yoga therapy on the voice quality of oral cancer patients. In the study, yoga therapy had no significant impact on the voice quality of those patients that received it (baseline data [all participants] ‐ 59.46 [M] ± 39.72 [SD]; endline data [control group] ‐ 66 (M) ± 35.29 (SD), *p*‐value = 0.884; endline data [experimental group] ‐ 58 (M) ± 23.40 (SD), *p*‐value = 0.995).

#### 3.6.3 General health

Only one study, by Bakshi and Goyal,^20^ explored the role of yoga therapy on the general health of oral cancer patients. In the study, yoga therapy had no significant impact on the general health of those patients that received it (baseline data [all participants] ‐ 18.08 [M] ± 6.1 [SD]; endline data [control group] ‐ 17.63 [M] ± 5.63 [SD], *p*‐value = 0.977; endline data [experimental group] ‐ 14 [M] ± 5.657 [SD], *p*‐value = 0.259). In fact, as shown in the statistics narrated in the preceding sentence, the endline data on general health, compared to that of the baseline, of both the control group and the experimental group decreased; however, the level of decrease was more pronounced in the experimental group than in the control group.

#### 3.6.4 Stress

Both studies explored the role of yoga on the perceived stress of oral cancer patients.^27^, ^28^ In the study by Bakshi and Goyal,^20^ yoga therapy had no significant impact on the stress level of those patients that received it, as there was no significant decrease in the level at endline (baseline data [all participants] ‐ 22.07 [M] ± 4.87 [SD]; endline data [control group] ‐ 21.13 [M] ± 8.0 [SD], *p*‐value = 0.915; endline data [experimental group] ‐ 17.17 [M] ± 4.6 [SD], *p*‐value = 0.181). However, it is noteworthy that the endline stress level among the intervention group is lower than that recorded among the control group.

However, in the study by Pattnaik et al.,^27^ it was observed that yoga training significantly decreased the stress levels of oral cancer patients (before intervention ‐ 48 [M] ± 0.99 [SD], after intervention – 37 [M] ± 5.2 [SD], *p*‐value < 0.001). Also, the study did not report any change of effect size across different subgroups.

#### 3.6.5 Anxiety level

Only one study, by Bakshi and Goyal,^20^ explored the role of yoga therapy on the anxiety level of oral cancer patients. In the study, yoga therapy significantly reduced the anxiety level of those patients that received it, as the reduction was more pronounced at endline, compared with the control group (baseline data [all participants] ‐ 54.15 [M] ± 10.63 [SD]; endline data [control group] ‐ 51.25 [M] ± 9.5 [SD], *p*‐value = 0.739; endline data [experimental group] ‐ 38.50 [M] ± 10.82 [SD], *p*‐value = 0.004).

#### 3.6.6 Mental well‐being

Only one study, by Bakshi and Goyal,^20^ explored the role of yoga therapy on the mental wellbeing of oral cancer patients. In the study, yoga therapy significantly increased the mental wellbeing of those patients that received it, as the improvement was more pronounced at endline, compared with the control group (baseline data [all participants] ‐ 37.69 [M] ± 10.41 [SD]; endline data [control group] ‐ 42.75 [M] ± 8.0 [SD], *p*‐value = 0.358; endline data [experimental group] ‐ 50.67 [M] ± 7.68 [SD], *p*‐value = 0.010).

#### 3.6.7 Blood pressure and pulse rate

Only one study, by Bakshi and Goyal,^20^ explored the role of yoga therapy on the systolic blood pressures (before yoga ‐ 109.1 [M] ± 24.35 [SD]; after yoga ‐ 105.4 [M] ± 14.22 [SD]; *p*‐value = 0.522), diastolic blood pressures (before yoga ‐ 69.75 [M] ± 12.23 [SD]; after yoga ‐ 71.54 [M] ± 25.02 [SD]; *p*‐value = 0.754) and pulse rates (before yoga ‐ 87.13 [M] ± 7.59 [SD]; after yoga ‐ 90.79 [M] ± 12.10 [SD], *p*‐value = 0.214) of the investigated oral cancer patients. In the study, yoga therapy did not cause any significant change in the these observed parameters (*p*‐values > 0.05).

## 4 DISCUSSION

Medical pluralism, a combination of western and traditional medicine is a global practice.^29^ Moreso for a degenerative disease, like cancer, people tend to use all sorts of traditional practices, especially in combination with modern medicine.^30^, ^31^ For instance, some medicinal herbs have been found to contribute positively to oral cancer treatment, which could signal opportunity for eventual development of new therapeutic strategies.^30^ It has also been noted that the use of traditional methods for oral cancer treatment was common.^31^ There are several conclusions that traditional medicine could be a significant and efficacious alternative in the treatment of oral cancers.^32^, ^33^ Hence, alternative medicine should be considered or integrated as part of national cancer management strategy.^33^ Yoga is considered a significant practice, which can impact positively on general wellbeing, and it is regarded as crucial in enhancing the quality of life in patients with cancer.^34^

This scoping review found that yoga could be effective in the management of stress among oral cancer patients. Other studies have found that yoga practice may assist cancer patients and survivors in managing symptoms such as anxiety, fatigue, insomnia depression, and pain.^34^, ^35^ Stress contributes adversely to the management of cancer leading to decreased quality of life. Yoga is a significant nonpharmacologic option to relieve stress and anxiety.^35^ The review underscores the role of yoga in improving the quality of life of advanced‐stage oral cancer patients. A study found that yoga improves relaxation and reduces stress.^27^, ^36^, ^37^ The overall effects account for improvement in routine activities which eventually increases the quality of life in cancer patients. Another study found the same effect of women with breast cancer that yoga practice is able to reduce stress and inflammation levels over time^37^ which impact positively on quality of life of population with cancer.

In addition, an integrative care approach that considers a nonpharmaceutical approach could help to reduce care cost while improving care outcomes and quality of life.^36^ Yoga could counterbalance the effects of radiotherapy with or without chemotherapy, which often result in some treatment complications. The effects of yoga cannot be compared with conventional treatment (radiotherapy and chemotherapy). Yoga presents a less complicated supplementary therapy, not a substitute, and there is no study recommending it alone in the care of oral cancer or any other cancers.

However, this scoping review has its limitation. The search strategy did not include gray literature and print‐only journals; hence, this may have limited the robustness of the search strategy. The exclusion of gray literature was deliberate because they are not peer reviewed. Also, print‐only journals were excluded due to inaccessibility.

## 5 CONCLUSION

Since yoga therapy significantly increased the mental wellbeing of the population with cancer, it is important to integrate yoga in the prevention and treatment of cancer. For a population with cancer, any therapy that would enhance their quality of life, especially without therapeutic complications should be embraced. Yoga targets fundamental concerns, especially stress and anxiety in cancer treatment. It is important that the health workers are familiarized with non‐pharmaceutical therapy such as yoga. It is imperative to consider yoga along with its potential benefits, and we recommend a gradual incorporation of yoga into oral cancer care.

## AUTHOR CONTRIBUTIONS

**Kehinde K. Kanmodi**: Conceptualization; data curation; formal analysis; funding acquisition; investigation; methodology; project administration; resources; software; supervision; validation; visualization; writing—original draft; writing—review & editing. **Ramat O. Braimah**: Resources; software; writing—original draft; writing—review & editing. **Jimoh Amzat**: Resources; writing—original draft; writing—review & editing. **Afeez A. Salami**: Data curation; investigation; methodology; resources; software; validation. **Lawrence A. Nnyanzi**: writing—review & editing.

## CONFLICT OF INTEREST STATEMENT

Kehinde Kazeem Kanmodi is an Editorial Board member of Health Science Reports. and a coauthor of this article. To minimize bias, they were excluded from all editorial decision‐making related to the acceptance of this article for publication. Other authors have no conflict of interest to declare.

## ETHICS STATEMENT

Not applicable. This study did not collect data from human or animal subjects but an open research repository.

## TRANSPARENCY STATEMENT

The lead author Kehinde Kazeem Kanmodi affirms that this manuscript is an honest, accurate, and transparent account of the study being reported; that no important aspects of the study have been omitted; and that any discrepancies from the study as planned (and, if relevant, registered) have been explained.

## Data Availability

Data sharing is not applicable to this article as no new data were created or analyzed in this study.

## 1

**Table A1:** Search string for PubMed database search.

Tag	Subject search	Search String
#1	Yoga	(((yoga[Title/Abstract]) OR (meditate[Title/Abstract])) OR (meditative[Title/Abstract])) OR (meditation[Title/Abstract])
#2	Oral cancer	((((((oral cancer[Title/Abstract]) OR (oral squamous cell carcinoma[Title/Abstract])) OR (oropharyngeal cancer[Title/Abstract])) OR (oral cavity cancer[Title/Abstract])) OR (mouth cancer[Title/Abstract])) OR (cancer of the lip[Title/Abstract])) OR (oral malignant neoplas*[Title/Abstract])
#3	#1 AND #2	(#1) AND (#2)

**Table A2:** Search string for SCOPUS database search.

Tag	Subject search	Search string
#1	Yoga	(TITLE‐ABS‐KEY (yoga) OR TITLE‐ABS‐KEY (meditate) OR TITLE‐ABS‐KEY (meditative) OR TITLE‐ABS‐KEY (meditation))
#2	Oral cancer	(TITLE‐ABS‐KEY (yoga) OR TITLE‐ABS‐KEY (meditate) OR TITLE‐ABS‐KEY (meditative) OR TITLE‐ABS‐KEY (meditation))
#3	#1 AND #2	(#1) AND (#2)

**Table A3:** Search string for other database (AMED – The Allied and Complementary Medicine Database; CINAHL Complete; CINAHL Ultimate; Dentistry and Oral Sciences Source; SPORTDiscus with Full Text; APA PsycInfo; APA PsycArticles; and Psychology and Behavioral Sciences Collection) search via EBSCO*Host* interface.

Tag	Subject search	Search String
S1	Yoga	AB Yoga OR AB meditate OR AB meditative OR AB meditation
S2	Oral cancer	AB Oral cancer OR AB oral squamous cell carcinoma OR AB oropharyngeal cancer OR AB oral cavity cancer OR AB mouth cancer OR AB cancer of the lip OR AB oral malignant neoplas*
S3	S1 AND S2	(S1) AND (S2)

**Table A4:** List of publications whose full texts were screened for inclusion into the review.

S/N	Citations	Decision status	
		Include	Exclude (with reasons)
1	Pattnaik SJ, Prasad RK, Jyotirmay, Pani P, Nishant, Kumar S. Yoga as a holistic approach for stress management in Oral Cancer patients. A prospective study. J Family Med Prim Care. 2020 Aug 25;9(8):4200‐4204. doi: 10.4103/jfmpc. jfmpc_612_20. PMID: 33110832; PMCID: PMC7586542.	Yes	
2	Olaku O, Conley BA, Ivy SP, McShane LM, Staudt LM, King SM, Sansevere M, Kim B, White JD. Survey of Lifestyle, Past Medical History and Complementary and Alternative Medicine Use Among Adult Patients Participating in the National Cancer Institute's Exceptional Responders Initiative. Transl Oncol. 2022 Nov;25:101484. doi: 10.1016/j. tranon.2022.101484. Epub 2022 Aug 6. PMID: 35944413; PMCID: PMC9365974.		Exclude (wrong study outcome)
3	C K S, Rao MV, Choudhary S, Singh OP. The healing potential of Draksha‐guduchyadi kavala in radiotherapy induced oral mucositis in non‐metastatic squamous cell carcinoma of head and neck: A comparative case study. J Ayurveda Integr Med. 2022 Apr‐Jun;13(2):100524. doi: 10.1016/j. jaim.2021.09.002. Epub 2021 Nov 26. PMID: 34844841; PMCID: PMC8728075.		Exclude (wrong study outcome)
4	Naing A, Stephen SK, Frenkel M, Chandhasin C, Hong DS, Lei X, Falchook G, Wheler JJ, Fu S, Kurzrock R. Prevalence of complementary medicine use in a phase 1 clinical trials program: the MD Anderson Cancer Center Experience. Cancer. 2011 Nov 15;117(22):5142‐50. doi: 10.1002/cncr.26164. Epub 2011 Apr 28. PMID: 21538342.		Exclude (wrong study outcome)
5	Chow WH, Chang P, Lee SC, Wong A, Shen HM, Verkooijen HM. Complementary and alternative medicine among Singapore cancer patients. Ann Acad Med Singap. 2010 Feb;39(2):129‐35. PMID: 20237735.		Exclude (wrong study outcome)
6	Kato I, Neale AV. Does use of alternative medicine delay treatment of head and neck cancer? A surveillance, epidemiology, and end results (SEER) cancer registry study. Head Neck. 2008 Apr;30(4):446‐54. doi: 10.1002/hed.20721. PMID: 18023030		Exclude (wrong study outcome)
7	Bakshi J, Goyal AK. Clinical yoga trial aim to improve quality of life at advanced stages of oral cancer. Sport Sci Health. 2021;17, 677‐685	Yes	
